# A longitudinal study of anxiety and cognitive decline in dementia with Lewy bodies and Alzheimer’s disease

**DOI:** 10.1186/s13195-016-0171-4

**Published:** 2016-01-26

**Authors:** Monica H. Breitve, Minna J. Hynninen, Kolbjørn Brønnick, Luiza J. Chwiszczuk, Bjørn H. Auestad, Dag Aarsland, Arvid Rongve

**Affiliations:** Department of Research and Innovation, Helse-Fonna HF Haugesund Hospital, Postbox 2170, 5504 Haugesund, Norway; Old Age Department, Clinic of Psychiatry, Helse-Fonna HF Haugesund Hospital, Postbox 2170, 5504 Haugesund, Norway; Faculty of Medicine, University of Bergen, Postbox 7804, 5020 Bergen, Norway; Department of Clinical Psychology, University of Bergen, Christies Gate 12, 5015 Bergen, Norway; NKS Olaviken Psychogeriatric Hospital, Ulriksdal 8, 5009 Bergen, Norway; TIPS – Centre for Clinical Research in Psychosis, Stavanger University Hospital, 4011 Stavanger, Norway; Neurological Department, Clinic of Medicine, Helse-Fonna HF Haugesund Hospital, Postbox 2170, 5504 Haugesund, Norway; Research Department, Stavanger University Hospital, Stavanger, Norway; Department of Mathematics and Natural Sciences, University of Stavanger, 4011 Stavanger, Norway; Centre for Age-Related Medicine, Stavanger University Hospital, Armauer Hansensvei 20, 4011 Stavanger, Norway; Division of Neurogeriatrics, Department of Neurobiology, Care Sciences and Society, Centre for Alzheimer Research, Karolinska Institutet, 141 57 Huddinge, Sweden; Network for Medical Sciences, University of Stavanger, 4036 Stavanger, Norway

**Keywords:** Alzheimer’s disease, Dementia with Lewy bodies, Dementia, Anxiety, BPSD, Longitudinal

## Abstract

**Background:**

Anxiety in dementia is common but not well studied. We studied the associations of anxiety longitudinally in Alzheimer’s disease (AD) and dementia with Lewy bodies (DLB).

**Methods:**

In total, 194 patients with a first-time diagnosis of dementia were included (*n* = 122 patients with AD, *n* = 72 patients with DLB). Caregivers rated the patients’ anxiety using the Neuropsychiatric Inventory, and self-reported anxiety was assessed with the anxiety and tension items on the Montgomery–Åsberg Depression Rating Scale. The Mini Mental State Examination was used to assess cognitive outcome, and the Clinical Dementia Rating (CDR)-Global and CDR boxes were used for dementia severity. Linear mixed effects models were used for longitudinal analysis.

**Results:**

Neither in the total sample nor in AD or DLB was caregiver-rated anxiety significantly associated with cognitive decline or dementia severity over a 4-year period. However, in patients with DLB, self-reported anxiety was associated with a slower cognitive decline than in patients with AD. No support was found for patients with DLB with clinical anxiety having a faster decline than patients with DLB without clinical anxiety. Over the course of 4 years, the level of anxiety declined in DLB and increased in AD.

**Conclusions:**

Anxiety does not seem to be an important factor for the rate of cognitive decline or dementia severity over time in patients with a first-time diagnosis of dementia. Further research into anxiety in dementia is needed.

## Background

Anxiety in dementia is common but not well studied. To our knowledge, no longitudinal studies of the associations between anxiety and cognitive decline have been done to date. The prevalence of anxiety in dementia is estimated to range from 25 % to 71 %, and generalized anxiety disorder seems to be the most common anxiety disorder [1]. People with co-morbid dementia and anxiety have more impairment in activities of daily living, reduced quality of life and more frequent nursing home admission than people with dementia [2–4]. They also use more health care services [5], and anxiety is associated with poorer relationships with caregivers and increased caregiver burden [6].

In elderly individuals with no dementia, both depression and anxiety have been shown to be early predictors of future cognitive decline [7, 8], and a more rapid progression in cognitive decline over time has been suggested, although the data are not conclusive [7, 9]. The risk of developing Alzheimer’s disease (AD) may be up to 30 times higher among persons with mild cognitive impairment (MCI) and anxiety [10]. A recently published study showed that anxiety symptoms in amnestic MCI predict conversion to AD to a greater degree than depression, memory loss or hippocampal cortex atrophy [11]. Moreover, it has been shown that anxiety is an independent predictor of future cognitive decline and not only via its relationship with depression, as often hypothesised in previous research [7, 12]. However, others have reported that anxiety in elderly people is not associated with increased risk of dementia or cognitive decline [13, 14].

Few studies of anxiety in dementia with Lewy bodies (DLB) have been done [15]. Ricci et al. [16] found that anxiety was more common in patients with DLB than in patients with AD, and this was later supported in a study by our group [17]. Anxiety also is more common in patients with DLB than in healthy control subjects and is reported to be a risk factor for development of DLB [18]. The severity of anxiety seems to be stable across stages of dementia, except for a decrease at the terminal stage [19, 20]. In a study of early-onset AD, the severity of anxiety was increasing over 3 years, but the patients were in different dementia severity stages at baseline [21].

Anxiety has overlapping symptoms with the dementia disease itself, as well as with other behavioural and psychological symptoms (BPSD) such as agitation and depression. According to Seignourel et al. [19], there is more support for that anxiety can be seen as a separate clinical entity, and is distinctive from agitation. Depression and anxiety have a high co-morbidity rate in both healthy elderly individuals and elderly persons with dementia [17, 22]. Therefore, depression must be controlled for to establish the independent contribution of anxiety as a predictor of cognitive decline.

Identifying predictors of cognitive decline is important, especially if an early intervention could reduce the speed of cognitive decline, both in MCI and in dementia. We investigated the association between anxiety and cognitive decline during 4 years of follow-up in people with AD and individuals with DLB.

## Methods

### Subjects

In total, 265 outpatients in clinics of old age psychiatry and geriatric medicine in western Norway with a first-time dementia diagnosis [Mini Mental State Examination (MMSE) score >15] were recruited starting in 2005 and followed annually. Patients with acute delirium or confusion, terminal illness, or current or previous bipolar disorder or psychotic disorder, or who were recently diagnosed with a major somatic illness, were excluded [23]. Follow-up examinations were conducted at the clinic or in nursing homes. The study protocol was approved by the Regional Committee for Medical and Health Research Ethics in Western Norway. Written informed consent was obtained from all participants in this study.

### Measures

#### Dementia diagnosis

The diagnosis of dementia was based on criteria set forth in the *Diagnostic and Statistical Manual of Mental Disorders, Fourth Edition*. AD was diagnosed according to the criteria of the National Institute of Neurological and Communicative Disorders and Stroke/Alzheimer’s Disease and Related Disorders Association [24]. DLB was diagnosed according to the revised consensus criteria [25]. For further information, see the publication by Aarsland et al. [23]. Two independent raters made the diagnoses, and so far the diagnosis has been neuropathologically confirmed in 36 cases.

#### Assessment of anxiety and depression

Symptoms of anxiety were rated on the basis of information obtained from a caregiver using Neuropsychiatric Inventory (NPI) [26] subscale E (Anxiety). On the NPI, symptoms are assessed for the previous 30 days, rating frequency (score 1–4) and severity (score 1–3). Frequency and severity are multiplied to obtain a sum score. A score above 4 is generally seen as clinically significant [27] and was used to identify DLB cases with clinical anxiety. In the present study, both categorical (caseness, presence of clinically significant anxiety) and dimensional scores were used.

Patients’ own perceptions of anxiety were measured with the anxiety item on the Montgomery–Åsberg Depression Rating Scale (MADRS) [28]. The MADRS items are rated from 0 to 6. The anxiety item (item 3) addresses “feelings of ill-defined discomfort, edginess, inner turmoil, mental tension mounting to either panic, dread or anguish” during the previous 3 days. Ratings of the MADRS and the NPI were conducted independently.

Caregiver-rated depression was measured with NPI subscale D (Depression), and self-reported depression was measured using MADRS.

#### Cognitive screening and dementia severity rating

The outcome measure—progression of dementia—was measured using two scales. The MMSE [29] is a brief test used to screen for cognitive impairment. The Clinical Dementia Rating (CDR) [30] is used to assess the severity of dementia on a scale from 0 to 3 (CDR-Global). The CDR scale also comprises six cognitive and practical domains (CDR boxes): Memory, Orientation, Judgement and Problem-Solving, Community Affairs, Home and Hobbies, and Personal Care.

#### Statistical analyses

Statistical analyses were performed using IBM SPSS 22.0 software (IBM, Armonk, NY, USA) and the R statistical software package [31]. Differences between AD and DLB at baseline were analysed with Mann–Whitney *U* tests because the variables were not normally distributed, and by Pearson χ^2^ tests for categorical data. Longitudinal analysis was done with linear mixed effects models using MMSE and CDR as dependent variables. For some outcomes, models with both random intercept and slope were used; for others, random intercept was sufficient according to model fit criteria. For longitudinal analyses of outcomes with very few categories, the different CDR (box) scores, the assumption of a normally distributed outcome poses a problem. To overcome this, we dichotomized the CDR scores and employed mixed effects logistic regression to analyse the probability of a CDR score of 2 or greater as a function of time, anxiety, depression and possibly other covariates.

## Results

**Fig. 1:**
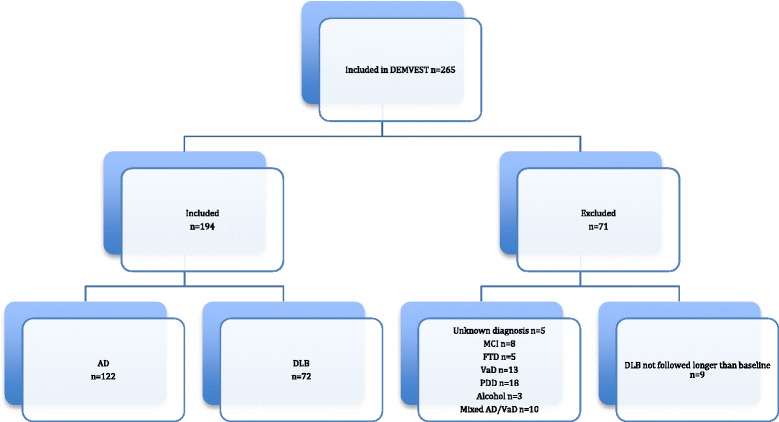
Flowchart of patient inclusion in the study. *AD* Alzheimer’s disease, *DemVest* Dementia Study of Western Norway, *DLB* dementia with Lewy bodies, *FTD* frontotemporal dementia, *MCI* mild cognitive impairment, *PDD* Parkinson’s disease dementia, *VaD* vascular dementia

Patients diagnosed with probable or possible AD (*n* = 122) and DLB (*n* = 72) were included in the analysis (see flowchart of study inclusion in Fig. 1). At baseline, patients with AD and patients with DLB did not differ in demographic and clinical variables, except that there were more women in the AD group and higher NPI total score in the DLB group (see Table 1).

**Table 1:** Participant characteristics at baseline

Characteristics	Total (*n* = 194)	AD (*n* = 122)	DLB (*n* = 72)	*p* Value*
Age, yr (SD)	75.8 (7.6)	75.7 (7.8)	76.0 (7.3)	0.951
Women, *n* (%)	122 (62.9)	90 (73.6)	32 (44.4)	<0.001
Education, yr (SD)	9.6 (2.9)	9.6 (3.0)	9.6 (2.9)	0.851
MMSE, mean (SD)	23.5 (2.7)	23.6 (2.3)	23.3 (3.1)	0.693
CDR-Global, median (IQR)	1.0 (0.5)	1.0 (0.5)	1.0 (0.5)	0.071
NPI anxiety, mean (SD)	1.8 (3.0)	1.5 (2.7)	2.2 (3.6)	0.197
NPI depression, mean (SD)	2.0 (2.6)	1.9 (2.5)	2.4 (2.7)	0.147
NPI total, mean (SD)	18.5 (17.3)	15.6 (16.0)	23.7 (18.2)	0.001
MADRS anxiety, median (IQR)	0 (2)	0 (2)	1 (2)	0.109
Clinically significant anxiety, % (median/IQR)	19.9	18.6 (6/4)	22.1 (8/8)	0.237
Non–clinically significant anxiety, % (median/IQR)	80.1	81.4 (0/0)	77.9 (0/1)	0.204

*Abbreviations*: *AD* Alzheimer’s disease, *DLB* Dementia with Lewy bodies, *MMSE* Mini Mental State Examination, *CDR* Clinical Dementia Rating, *IQR* interquartile range, *NPI* Neuropsychiatric Inventory, *MADRS* Montgomery–Åsberg Depression Rating Scale, *SD* standard deviation

*Differences between the AD and DLB groups were analysed using the Mann-Whitney *U* test and Pearson’s *χ* ^2^ test

We found no significant interaction between time and anxiety—neither caregiver-rated nor self-reported anxiety—on the MMSE, CDR-Global or CDR boxes.

There was no significant interaction between diagnostic group, time and anxiety (caregiver-rated or self-reported) on the MMSE, CDR-Global or CDR boxes, except for an interaction between diagnostic group, time and MMSE for self-reported anxiety (*p* = 0.039), even after controlling for depression and/or total MADRS score, indicating that anxiety in DLB was associated with a slower cognitive decline than in AD.

There was no significant interaction in patients with DLB between clinical significant anxiety at baseline and time on the MMSE, CDR-Global or CDR boxes.

**Fig. 2:**
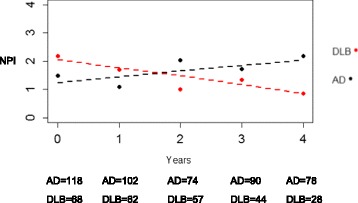
Anxiety as evaluated with the Neuropsychiatric Inventory (NPI) over the course of 4 years in patients with Alzheimer’s disease (AD) and patients with Lewy bodies (DLB)

There was a significant interaction between diagnostic group and time for caregiver-reported anxiety (*p* = 0.002). Patients with DLB had a higher level of anxiety at baseline than patients with AD, but the anxiety level was reduced over time. This was contrary to patients with AD, among whom the level of anxiety increased over time, adjusted for CDR-Global and age (see Fig. 2). There was no significant interaction between diagnostic group and time for self-reported anxiety.

## Discussion

In this longitudinal cohort study, we analysed the association of anxiety and cognition over 4 years in people with AD and individuals with DLB. We found no clear indication that anxiety was associated with a faster decline in cognition or dementia severity in either the total sample or in DLB compared with AD, or between patients with DLB with or without clinical anxiety. The only observation was that anxiety in DLB was associated with a slower cognitive decline than in AD.

The level of caregiver-reported anxiety was different between patients with AD and patients with DLB over time. During the observation period over 4 years, the level of anxiety in patients with DLB was declining, which was the opposite of an increasing anxiety level in patients with AD. Self-reported anxiety was not found to be different over time.

The observed decline of anxiety in DLB over time could be due to treatment of anxiety-provoking hallucinations or to increased insight and learning how to interpret the hallucinations as perceptual errors so that they become pseudo-hallucinations.

Both psychological and biological mechanisms probably cause the anxiety that occurs in patients with dementia. Patients with dementia experience less coping in daily living, lose overview and control, and might fear for the future [19, 32]. In addition, atrophy and/or dysfunctions in the limbic structures may make them less capable of coping with fear and anxiety [18]. Higher levels of cortisol due to stress can also cause hippocampal atrophy [11]. Furthermore, patients with MCI and anxiety have pathological AD markers in the cerebrospinal fluid which are not found in patients with depression and MCI, which may also support a role of biological mechanisms [33].

Even though we did not find that anxiety was associated with the rate of cognitive decline or dementia severity, it is important to focus on anxiety to avoid negative consequences for patients and their caregivers, as mentioned above. In dementia care, anxiety should be screened for and, when required, thoroughly mapped before intervention.

Some limitations of our study should be noted. We used NPI as the main anxiety measure, which is an indirect way to measure anxiety via relatives or nursing home personnel. This is a commonly used and validated measure in dementia research, but it may not have satisfactory psychometric properties for measuring anxiety [19]. In factor analysis of NPI, anxiety and depression often load on the same factor. A majority of the studies have included only patients with AD, and we found few published studies that included patients with DLB or analysed them as a subgroup [34, 35]. In our study, the correlations between anxiety and other subtests from the NPI at baseline were low, both in the total sample and in AD and DLB, but in the longitudinal analysis we controlled for depression to ensure that an observed effect from anxiety was not due to a BPSD subsyndrome.

It has been recommended that, when studying anxiety in people with dementia, information should be collected from multiple sources because the caregivers can report verbalized or behavioural signs of anxiety but not the internalized symptoms [19]. We therefore used one item on MADRS to measure self-reported anxiety, and it is based on the reliability of the patients’ answer. Bradford et al. [36] suggested that people with mild to moderate dementia can give reliable self-reports of anxiety symptoms, with validity comparable to reports obtained from caregivers. In our sample, after 4 years, 34 % had a CDR score of 3, which indicates severe dementia and can challenge the reliability of the score. In addition, assessing anxiety with only one item from a depression scale is not optimal and limits the reliability of the findings. Observations based only on this score which are not in accordance with established research should therefore be carefully weighed.

The MMSE and the CDR scale were used as outcome measures. MMSE has been criticized for not being able to adequately monitor cognitive decline in pure DLB [37]. However, the MMSE was found to be sensitive for cognitive changes in Parkinson’s disease, which shares many features with DLB [38]. CDR is designed for the phenotypes of AD, and less is known about the psychometric properties when addressing DLB.

There were also missing data in our analysis, due both to the inevitable facts that the dementia was progressing and the patients were not able to participate in the assessments, and also because of natural mortality during follow-up. The most appropriate analysis, linear mixed effects, was used to minimize this problem. There is still a possibility that the null hypothesis was supported because of inadequate power. Unfortunately, the dataset was not large enough to perform joint analysis.

The strengths of our study are that it include a relatively large number of patients with DLB and that they were followed annually until death. Patients were included in the study according to the latest DLB consortium criteria; they were thoroughly evaluated at baseline; some also underwent ioflupane single-photon emission computed tomography; and the diagnosis of the patients was revised after 2 and 5 years by an expert panel. Post-mortem autopsies of 36 patients to date have confirmed the clinical diagnosis, with the exception of two false-positive cases and one false-negative case for DLB.

## Conclusions

In this study, anxiety was not associated with the dementia progression rate in AD or DLB. Anxiety gradually decreased over 4 years in DLB but gradually increased in AD. More studies are needed.

## Abbreviations

AD: Alzheimer’s disease
BPSD: behavioural and psychological symptoms
CDR: Clinical Dementia Rating
DemVest: Dementia Study of Western Norway
DLB: dementia with Lewy bodies
FTD: frontotemporal dementia
IQR: interquartile range
MADRS: Montgomery–Åsberg Depression Rating Scale
MCI: mild cognitive impairment
MMSE: Mini Mental State Examination
NPI: Neuropsychiatric Inventory
PDD: Parkinson’s disease dementia
SD: Standard deviation
VaD: vascular dementia
